# Why are CGRP monoclonal antibodies not yet the first line treatment in migraine prevention?

**DOI:** 10.1590/0004-282X-ANP-2022-S125

**Published:** 2022-08-12

**Authors:** Caio Vinicius de Meira Grava Simioni

**Affiliations:** 1 Universidade de São Paulo, Faculdade de Medicina, Hospital das Clínicas, Departamento de Neurologia, São Paulo, SP, Brazil.; 2 Hospital A. C. Camargo Cancer Center, Departamento de Neurologia Adulto, São Paulo, SP, Brazil.

**Keywords:** Antibodies, Monoclonal, Costs and Cost Analysis, Efficacy, Health Services Needs and Demand, Migraine Disorders, Headache, Pharmaceutical Preparations, Prescriptions, Safety, Surveillance, Therapeutics, Anticorpos Monoclonais, Custos e Análise de Custo, Eficácia, Cefaleia, Necessidades e Demandas de Serviços de Saúde, Transtornos de Enxaqueca, Preparações Farmacêuticas, Prescrições, Segurança, Vigilância, Terapêutica

## Abstract

Migraine is a prevalent disorder and a cause of high disability, influenced by modifiable and non-modifiable risk factors. Comorbid and psychiatric illnesses are prevalent in migraine patients and should be considered when choosing preventive drugs. There have been unforeseen problems with the use of preventive treatment of migraine with oral drugs, mainly due to side-effects that cannot be tolerated and lack of efficacy, leading to high discontinuation rates. Anti-CGRP monoclonal antibodies (mAbs) have shown better tolerance profiles, based on the low dropout rates in clinical trials due to adverse events. First-line therapy is a term most expressed in some medical specialties that adopt standardized protocol treatments and may not be suitable for treating migraine. Regarding efficacy, mAbs don’t seem to perform much better than the current prophylactic oral drugs in reduction of monthly migraine days compared to placebo. Monoclonal antibodies against CGRP pathway have been prescribed recently, which raises some concern about their safety in the long term. Only side effects observation will confirm whether CGRP blockade causes susceptibility to severe side-effects, at least to specific subpopulations. CGRP may play a role in regulating uteroplacental blood flow and myometrial and uterine relaxation, as well as blood pressure control, raising the suspicion that its blockade could cause complications during pregnancy. Recent guidelines retain the recommendation of starting preventive treatment of migraine with oral drugs. Both the fact that it is new and costs are the reason why guidelines recommend the prescription of mAbs only after failure of at least two oral drugs.

## RESUMO

A migrânea é uma condição prevalente e motivo de grande incapacidade, influenciada por fatores de risco modificáveis e não-modificáveis. Comorbidades e doenças psiquiátricas são prevalentes em doentes com migrânea e devem ser levadas em consideração na escolha do tratamento profilático com medicações orais. Os anticorpos monoclonais anti-CGRP possuem melhor perfil de tolerabilidade, baseando-se nos baixos indicadores de desistência devido a efeitos colaterais em ensaios clínicos. O termo “tratamento de primeira linha” é muito utilizado em algumas especialidades médicas que adotam protocolos de tratamento padronizados e pode não ser adequado à migrânea. Quanto à eficácia, os anticorpos monoclonais não possuem desempenho muito superior que os medicamentos profiláticos orais atuais no tocante à redução de dias de migrânea por mês, quando comparados ao placebo. Os anticorpos monoclonais anti-CGRP são recentes no mercado, o que leva a suspeitas quanto à sua segurança a longo prazo. Apenas a vigilância de efeitos adversos confirmará se o bloqueio da via do CGRP não leva à suscetibilidade de efeitos colaterais graves, ao menos em subpopulações específicas. O CGRP pode ter um papel na regulação do fluxo sanguíneo uteroplacentário, bem como no relaxamento do miométrio e do útero e controle da pressão arterial, levando à possibilidade de que o seu bloqueio poderia causar complicações durante a gestação. Guidelines recentes recomendam o início do tratamento preventivo da migrânea com drogas orais. Tanto a precocidade quanto os elevados custos são a razão porque os guidelines orientam a prescrição de anticorpos monoclonais após falha a pelo menos duas medicações orais.

## INTRODUCTION

Migraine is a common neurologic disorder and the second cause of disability worldwide, leading to more disability than all other neurologic diseases^1^. The prevalence of migraine is roughly 12% (18% in women and 6% in men) and approximately 2.5% of people with episodic migraine progress to chronic migraine, whose prevalence is estimated at 1-2% of the general population^2^. Migraine ranked second in prevalence among non-communicable diseases, and as the highest cause of disability among adults in Brazil^3^, where care is provided by either public or private health sectors. The public health sector is very under-resourced, leading to misdiagnosis and, consequently, to inappropriate treatment^4^, which should be improved by headache public policies.

## ARE THERE UNFORESEEN PROBLEMS IN MIGRAINE PREVENTIVE TREATMENT?

Migraine manifests as a continuum, from episodic to chronic migraine^5^. Some modifiable risk factors such as frequency of attacks, obesity, medication overuse, stressful life events, caffeine overuse and snoring, as well non-modifiable risk factors such as age, gender, and socioeconomic status, are related to worsening of migraine over time^6^. Therefore, migraine cannot be considered strictly as a monomorphic disease, but as a spectrum of clinical manifestations influenced by genetic factors and lifestyle. Because comorbid medical and psychological illnesses are prevalent in patients who have migraine, comorbidities must be considered when choosing preventive drugs, particularly in chronic migraine^7^ ^,^ ^8^. However, until around 2015, there were unforeseen problems as to preventive treatment of migraine with oral drugs, mainly due to lack of tolerance of side-effects and lack of efficacy, leading to high discontinuation rates^9^. On the other hand, anti-CGRP monoclonal antibodies (mAbs) have shown better tolerance profiles, based on the low dropout rates due to adverse events in clinical trials, when compared to previous preventive medications. Furthermore, central nervous system specific adverse events have not been described with mAbs to date, contrasting with oral treatments^10^. In view of this new form of therapy, one question arises: “Can CGRP monoclonal antibodies be first line therapy in migraine prophylaxis?”. Some caveats to this will be explored below.

## FIRST LINE THERAPY REFERRING TO MIGRAINE AND CURRENT TREATMENTS

“First line therapy” is a current definition of the best treatment for a given disease and clearly implies better outcomes and lower adverse event rates when compared to other available treatments^11^. The term is most used in particular medical specialties (e.g., oncology), which adopt standardized protocol treatments derived from evidence-based medicine. In fact, alongside clinical examination, imaging, laboratory and other biological markers there is a growing importance in medical decision-making^12^. Bearing this in mind, could it be applied to migraine? So far, after ruling out secondary headaches, migraine diagnosis is fully dependent on medical skills and to date there are no biological markers to ensure “the best” treatment option on an individual basis^13^. Thus, for instance, mAbs can be the best option for a patient with a high adverse events profile, whereas topiramate or propranolol, for example, could be useful for patients who have not experienced side-effects and who possibly would not complain of them during treatment. These patients could also benefit from treatment of other medical conditions, such as systemic arterial hypertension, essential tremor, depression, epilepsy, etc., which are not considered with mAbs^14^. In summary, oral drugs are helpful in treating migraine and its comorbidities, while mAbs are targeted only at migraine treatment (despite subgroup analysis with comorbid depression having responded to the blockade of CGRP pathway)^15^.

## CURRENT TREATMENTS AND MABS EFFICACY

Regarding efficacy, mAbs don’t seem to perform much better than the current prophylactic oral drugs (at most, slightly better) in reduction of monthly migraine days compared to placebo. Table 1 shows this finding with mAbs, Level of evidence “A” oral preventive drugs and onabotulintoxin A^16^ ^-^ ^18^.

**Table 1.** Change in monthly migraine days.

Treatment	Mean difference (95% CI)
Eptinezumab vs. Placebo	-1.43 [-2.59, -0,36]
Erenumab vs. Placebo	-1.61 [-2.40, -0.84]
Fremanezumab vs. Placebo	-2.19 [-3.15, -1.25]
Galcanezumab vs. Placebo	-2.10 [-2.76, -1.45]
Topiramate vs. Placebo	-1.40 [-2.20, -0.50]
Divalproex sodium vs. Placebo	-1.50 [-2.20, -0.76]
Propranolol vs. Placebo	-1.00 [-2.10, -0.39]
Metoprolol vs. Placebo	-0.94 [-1.40, -0.46]
Onabotulintoxin A vs. Placebo*	-2.00 [-2.67, -1.27]

*Only chronic migraine; Crl: credible interval.

## LONG-TERM SAFETY

Long-term safety is also relevant when selecting some first line therapy. One of the greatest advantages of mAbs is the low adverse effect rates, (most of them of mild intensity), leading to better adherence to treatment^19^. Nevertheless, monoclonal antibodies against CGRP pathway have been prescribed recently, which raises some concern about their safety in the long term. Conversely, oral treatments have been prescribed for decades and all their pros and cons are well known at this point. In fact, they have withstood the test of time, despite some of them having been discontinued by virtue, mainly, of lack of tolerance of side-effects (e.g.: pizotifen and metisergide)^20^. Experience with earlier treatments causes confidence as to short and long-term side effects, something not yet proven with mAbs.

## CGRP IS INVOLVED IN OTHER SYSTEMS

The function of CGRP in both peripheral and enteric nervous systems is well established - it is involved not only in migraine, but also other physiological processes and in homeostatic responses during pathophysiological conditions. CGRP is present in nerve fibers that innervate blood vessels and the heart, acts in the regulation of blood pressure and may have a role in maintenance of cardiovascular homeostasis during ischemic events and tissue remodeling in pulmonary hypertension. CGRP is also involved in inflammatory processes and facilitation of wound healing. In addition, it is found in the anterior pituitary, possibly influencing the regulation of hypothalamus-pituitary tract functions. To date, there are no reports on safety issues related to these functions, and only observation of side effects will confirm whether or not CGRP blockade causes susceptibility to severe side-effects, at least to specific subpopulations^21^.

## CONCERNS WITH WOMEN IN CHILDBEARING AGE

Migraine mostly affects women during their fertile years. There are no controlled studies of mAbs involving pregnant or lactating women. A limited number of safety reports have indicated no specific maternal toxicities, major birth defects or increased reporting of spontaneous abortions when mAbs is administered during pregnancy, exposure shortly prior to pregnancy or breast-feeding^22^. CGRP may play a role in regulating uteroplacental blood flow and myometrial and uterine relaxation, as well blood pressure control, raising the suspicion that its blockade could cause gestational hypertension, preeclampsia or eclampsia^23^. Therefore, a woman of childbearing age should be advised as to the lack of safety data of mAbs during pregnancy and lactation and, if she is currently on mAb and wishes to get pregnant, be told that she should withdraw the treatment and wait a few months, when one may assume that mAb has been entirely cleared.

## MIGRAINE TREATMENT GUIDELINES

Finally, recent guidelines retain the recommendation for starting the preventive treatment of migraine with oral drugs. The American Headache Society Consensus Statement criteria for initiating mAbs are^24^:

- Prescribed by a licensed clinician;
- Patient is at least 18 years of age;
- Diagnosis of ICHD-3 migraine with or without aura (4-7 monthly migraine days) or
- Diagnosis of ICHD-3 migraine with or without aura (8-14 monthly migraine days) or
- Diagnosis of ICHD-3 chronic migraine (≥15 monthly headache days, at least 8 days with migraine features).

And inability to tolerate (due to side effects) or inadequate response to an 8-week trial of two or more of the following:

- Topiramate
- Divalproex sodium/valproate sodium
- Beta-blocker: metoprolol, propranolol, timolol, atenolol, nadolol
- Tricyclic antidepressant: amitriptyline, nortriptyline
- Serotonin-norepinephrine reuptake inhibitor: venlafaxine, duloxetine
- Other Level A or B treatments (established efficacy or probably effective) according to AAN scheme for classification of evidence.

Inability to tolerate or inadequate response to a minimum of two quarterly injections (6 months) of onabotulinumtoxinA (only for chronic migraine).

The recommendations of the European Headache Federation published in 2019 about the use of mAbs in subjects with migraine are^25^:

- When should treatment with anti-CGRP monoclonal antibodies be offered to patients with migraine?

In patients with episodic or chronic migraine who have failed at least two of the available medical treatments or who cannot use other preventive treatments because of comorbidities, side effects or poor compliance, we suggest the use of erenumab, fremanezumab, or galcanezumab.

The Consensus of the Brazilian Headache Society on the treatment of chronic migraine has not incorporated guidelines on CGRP mAbs, because they were not yet available in Brazil in 2019^26^.

Costs of CGRP mAbs are not entirely known but, presumably, these are higher than oral treatments that are available. Further studies could provide information about the economic impact of mAbs, taking into account direct and indirect costs related to migraine^25^.

In conclusion, undoubtedly CGRP monoclonal antibodies are a breakthrough in migraine preventive treatment; their specific mode of action, rapid response and safety profile to date show they are innovative and could soon be the first line therapy for episodic and chronic headache. Nevertheless, the concept of “first line treatment” is not suitable for migraine, since doctors should practice clinical examination skills to make a proper diagnosis and “a tailor-made” treatment, taking into account age, comorbidities, pregnancy risk, previous treatments, etc. Medical and psychiatric comorbid conditions are addressed by current oral preventive drugs, but not by mAbs.

 As with all new therapies, mAbs have not been subject to the test of time regarding long-term side effects. Subpopulations could still be at risk while pregnant or breast-feeding women, alcohol or drug abusers, people with cardio and cerebrovascular diseases as well as severe mental disorders are the most relevant of these^25^. Finally, pharmacoeconomic factors play a decisive role in governmental decisions; costs of mAbs are still a barrier to using them as first choice therapy for migraine prophylaxis. Both the fact that they are new and costs are the reason why guidelines only recommend the prescription of mAbs after failure of at least two oral drugs^10^.

## References

[B1] GBD 2016 Disease and Injury Incidence and Prevalence Collaborators (2017). Global, regional, and national incidence, prevalence, and years lived with disability for 328 diseases and injuries for 195 countries, 1990-2016: a systematic analysis for the Global Burden of Disease Study 2016. Lancet.

[B2] Burch RC, Buse DC, Lipton RB (2019). Migraine: epidemiology, burden, and comorbidity. Neurol Clin.

[B3] Peres MFP, Queiroz LP, Rocha-Filho PS, Sarmento EM, Katsarava Z, Steiner TJ (2019). Migraine: a major debilitating chronic non-communicable disease in Brazil, evidence from two national surveys. J Headache Pain.

[B4] Krymchantowski AV, Jevoux CC (2015). The pharmacological treatment of migraine in Brazil. Headache.

[B5] Bigal ME, Lipton RB (2008). Clinical course in migraine: conceptualizing migraine transformation. Neurology.

[B6] Bigal ME, Lipton RB (2006). Modifiable risk factors for migraine progression. Headache.

[B7] Weatherall MW (2015). The diagnosis and treatment of chronic migraine. Ther Adv Chronic Dis.

[B8] Silberstein SD (2009). Preventive migraine treatment. Neurol Clin.

[B9] Blumenfeld AM, Bloudek LM, Becker WJ, Buse DC, Varon SF, Maglinte GA (2013). Patterns of use and reasons for discontinuation of prophylactic medications for episodic migraine and chronic migraine: results from the second international burden of migraine study (IBMS-II). Headache.

[B10] Raffaelli B, Neeb L, Reuter U (2019). Monoclonal antibodies for the prevention of migraine. Expert Opin Biol Ther.

[B11] National Cancer Institute (2011). Definition of first-line therapy - NCI Dictionary of Cancer Terms.

[B12] Price CP (2000). Evidence-based laboratory medicine: supporting decision-making. Clin Chem.

[B13] Buse DC, Lipton RB (2008). Facilitating communication with patients for improved migraine outcomes. Curr Pain Headache Rep.

[B14] Silberstein SD (2015). Preventive migraine treatment. Continuum (Minneap Minn).

[B15] Lipton RB, Cohen JM, Galic M, Seminerio MJ, Yeung PP, Aycardi E (2021). Effects of fremanezumab in patients with chronic migraine and comorbid depression: subgroup analysis of the randomized HALO CM study. Headache.

[B16] Wang X, Chen Y, Song J, You C (2021). Efficacy and safety of monoclonal antibody against calcitonin gene-related peptide or its receptor for migraine: a systematic review and network meta-analysis. Front Pharmacol.

[B17] Loder E, Rizzoli P (2018). Pharmacologic prevention of migraine: a narrative review of the state of the art in 2018. Headache.

[B18] Aurora SK, Winner P, Freeman MC, Spierings EL, Heiring JO, DeGryse RE (2011). OnabotulinumtoxinA for treatment of chronic migraine: pooled analyses of the 56-week PREEMPT clinical program. Headache.

[B19] Do TP, Guo S, Ashina M (2019). Therapeutic novelties in migraine: new drugs, new hope?. J Headache Pain.

[B20] Sprenger T, Viana M, Tassorelli C (2018). Current prophylactic medications for migraine and their potential mechanisms of action. Neurotherapeutics.

[B21] Deen M, Correnti E, Kamm K, Kelderman T, Papetti L, Rubio-Beltrán E (2017). Blocking CGRP in migraine patients - a review of pros and cons. J Headache Pain.

[B22] Tinsley A, Rothrock JF (2021). Safety and tolerability of preventive treatment options for chronic migraine. Expert Opin Drug Saf.

[B23] Dodick DW (2019). CGRP ligand and receptor monoclonal antibodies for migraine prevention: evidence review and clinical implications. Cephalalgia.

[B24] Ailani J, Burch RC, Robbins MS, Board of Directors of the American Headache Society (2021). The American Headache Society Consensus Statement: update on integrating new migraine treatments into clinical practice. Headache.

[B25] Sacco S, Bendtsen L, Ashina M, Reuter U, Terwindt G, Mitsikostas DD (2019). European headache federation guideline on the use of monoclonal antibodies acting on the calcitonin gene related peptide or its receptor for migraine prevention. J Headache Pain.

[B26] Kowacs J, Roesler CAP, Piovesan ÉJ, Sarmento EM, Campos HC, Maciel JA (2019). Consensus of the Brazilian Headache Society on the treatment of chronic migraine. Arq Neuropsiquiatr.

